# Supplementary material for: The Evolution and Characterization of the RNA Interference Pathways in Lophotrochozoa
Source: Genome Biol Evol. 2024 May 7;16(5):evae098. doi: 10.1093/gbe/evae098 (PMC11114477; doi:10.1093/gbe/evae098)
Supplement: evae098_Supplementary_Data [file evae098_supplementary_data.zip › Supplementary_materials.zip!/selected_miRNAs/NoGe_selected_miRNAs/known_miRNA_families_pdf_files/NMRB01000380.1_Notospermus_geniculatus_strain_Ushimado_scaffold380,_whole_genome_shotgun_sequence_11924.pdf]

Provisional ID	:	NMRB01000380.1_Notospermus_geniculatus_strain_Ushimado_scaffold380,_whole_genome_shotgun_sequence_11924
Score total	:	42727.8
Score for star read(s)	:	3.9
Score for read counts	:	42718.9
Score for mfe	:	2
Score for randfold	:	0
Score for cons. seed	:	3
Total read count	:	83803
Mature read count	:	83505
Loop read count	:	0
Star read count	:	298

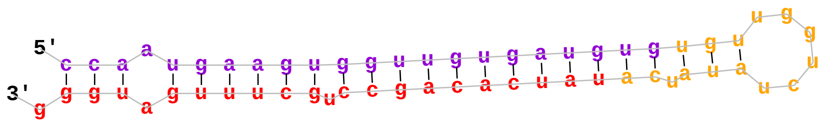

Genome coordinates: NMRB01000380.1 234397-234456

pre-miRNA sequence:
CCAAUGAAGUGGUUGUGAUGUGCCAAUGAAGUGGUUGUGAUGUGUGUUGGUCUAUAUCAUAUCACAGCCUGCUUUGAUGGG

Mature sequence: UAUCACAGCCUGCUUUGAUGGG

Aligned mature sequence of the *N. geniculatus* pre-miRNA with the top 5 hits from the BLAST search against the pre-miRNA mirGeneDB set:

```
                            ******** ********* *
Nge_Mir-2-o71              uaucacagccugcuuugauggg-----
Tca-Mir-2-P4-v2_3p         --ucacagccagcuuugaugagcgcaa
Lgi-Mir-2-o15_3p           uaucacagccagcuuugaugagu----
Lan-Mir-2-o78_3p           uaucacagccugcuuugaugagc----
Lan-Mir-2-o73_3p           uaucacagccugcuuugaugaga----
Lan-Mir-2-o71_3p           uaucacagccugcuuugaugagc----
```
